# AKAP12 and AKAP5 form higher-order hetero-oligomers

**DOI:** 10.1186/1750-2187-6-8

**Published:** 2011-08-10

**Authors:** Shujuan Gao, Hsien-yu Wang, Craig C Malbon

**Affiliations:** 1 Department of Pharmacology, Health Sciences Center, School of Medicine, State University of New York at Stony Brook, Stony Brook, NY 11794-8651 USA; 2 Department of Physiology & Biophysics, Health Sciences Center, School of Medicine, State University of New York at Stony Brook, Stony Brook, NY 11794-8661 USA

**Keywords:** AKAP5, AKAP12, gravin, SSECKS, protein kinase A, scaffold, beta-adrenergic receptor, homo-oligomer, hetero-oligomer, oligomerization

## Abstract

**Background:**

The family of A-kinase-anchoring proteins, AKAPs, constitutes a group of molecular scaffolds that act to catalyze dynamic interactions of protein kinase A, protein kinase C, tyrosine kinases, G-protein-coupled receptors and ion channels. AKAP5 (MW ~47 kDa) and AKAP12 (MW ~191 kDa) homo-oligomerize, but whether or not such AKAPs can hetero-oligomerize into supermolecular scaffolds of increased complexity is unknown.

**Results:**

Affinity chromatography using immobilized AKAPs as "bait" demonstrates unequivocally that AKAP5 and AKAP12 do form minimally hetero-dimers. Steric-exclusion chromatography of AKAP5 and AKAP12 mixtures revealed the existence of very large, supermolecular complexes containing both AKAPs. Docking of AKAP5 to AKAP12 was increased 4-fold by beta-adrenergic agonist stimulation. Overexpression of AKAP12 was found to potentiate AKAP5-mediated Erk1/2 activation in response to stimulation with beta-adrenergic agonist.

**Conclusion:**

AKAP5 and AKAP12 are capable of forming hetero-oligomeric supermolecular complexes that influence AKAP locale and function.

## Background

Scaffold proteins have emerged as essential elements of cell signaling, providing docking sites at which protein kinases, phosphoprotein phosphatases, G-protein-linked receptors/ion channels can interact. An important subset of scaffold molecules possesses a docking site for the regulatory subunits (*i.e*., RI/RII) of cyclic AMP-dependent protein kinase A (PKA, A-kinase), termed A-kinase-anchoring proteins (AKAP) intimately involved in cellular signaling [1-4]. AKAPs dock PKA, acting as well as molecular "tool boxes" reflecting multivalency and the ability to dock other signaling proteins, including a full range of protein kinases (e.g., PKA, protein kinase C [5-8], and the tyrosine kinases [9]), phosphoprotein phosphatases (e.g., protein phosphatase 2B (PP2B) [5,10], cyclic AMP phosphodiesterases (e.g., PDE4) [11-14], adaptor molecules [11,13,15,16], ion channels [17-20], and members of the superfamily of G protein-coupled receptors (GPCR) [21-23]. AKAP5 and AKAP12, for example, associates with the prototypic GPCR, the β_2_-adrenergic receptor [23]. To what extent these AKAPs associate with other members of the GPCR superfamily is not known. The AKAPs that do dock GPCRs have been one of the major foci of AKAP research [23-26].

In 2003 we first reported the oligomerization of AKAPs [27], noting that AKAP12 oligomers were SDS-resistant and could only be disassembled in the presence of a chaotropic agent, such as 8 M urea. More recently, oligomerization has been reported for AKAP5 [28,29], although AKAP5 oligomers are not SDS-resistant. AKAP5 oligomers display MW on wide-bore steric exclusion chromatography indicative of homo-oligomers of dimers and tetramers [28]. That both AKAP5 and AKAP 12 were capable of forming large, homo-oligomeric complexes (e.g., dimers and tetramers) provoked our interest in interrogating the exciting possibility that AKAP scaffolds might form hetero-oligomers, capable of extending the functional repertoire of docking proteins assembled by each [28]. Herein, we probe further these two members of the class of GPCR-associated AKAPs and address the extent to which these proteins are capable of forming AKAP hetero-oligomers. Both AKAP5 and AKAP12 are predicted to be more than 85% natively unordered [10], based upon primary sequence information alone. The current work is the first to report that both AKAP5 and AKAP12 form hetero-oligomers, i.e., large supermolecular assemblies of AKAP5-AKAP12 that are functionally significant. Thus AKAP-AKAP docking adds a new dimension on how members of this class of scaffold molecules function in cell signaling.

## Materials and methods

### Antibodies

Mouse anti-AKAP5, anti-pERK monoclonal antibody, rabbit anti-ERK, goat anti-mouse IgG-HRP, and goat anti-rat IgG-HRP were purchased from Santa Cruz Biotechnology (Santa Cruz, CA). Mouse anti-AKAP12 monoclonal antibody was purchased from Abcam (Cambridge, MA). Mouse anti-GFP, Rat anti-HA antibody and HA-agarose beads are products of Roche (Indianapolis, IN).

### Cell lines

The human epithelial carcinoma cell line A431 [30,31] and human embryonic kidney cell line HEK293 [14,23,24,32] were obtained from ATCC (Bethesda, MD) and cultured in Dulbecco's modified Eagle's medium (DMEM) containing 10% fetal bovine serum in a humidified atmosphere containing 5% CO_2 _at 37°C. Confluent cells were treated with 10 μM isoproterenol (Iso) in DMEM, for indicated times.

### Plasmids and transfection

pcDNA3.1 vector carrying HA-tagged AKAP12, HA-AKAP12 (1-362), HA-AKAP12 (1-652), HA-AKAP12 (554-938), HA-AKAP12 (1-938), and HA-AKAP12 (840-1782) were constructed as described previously [27]. pCMV-HA vector carrying AKAP5, His-tagged AKAP12(840-1782), AKAP12(1-840) and AKAP5 were constructed as described previously[28]. A431 or HEK293 cells were transfected with plasmid DNA using *ExpressFect*^® ^(Denville Scientific, Metuchen, NJ), according to the manufacturer's instructions. A431 cells stably expressing AKAP79-GFP or AKAP12-GFP were established using G418 at 400 μg/ml and maintained at 100 μg/ml.

### Purification of His-tagged AKAP5 and AKAP12

His-tagged proteins expressed in *E. coli *were purified by using Ni-NTA matrix (Qiagen, Valencia, CA), according to the manufacturer's protocol.

### Crosslinking of AKAPs to CNBr-activated Sepharose 4B

Purified His-tagged AKAP12 (1-840), His-tagged-AKAP12 (840-1782) were cross-linked to CNBr-activated Sepharose 4B (GE Healthcare), as described in the product manual.

### Steric-exclusion chromatography of AKAP supermolecular complexes

Purified His-tagged AKAP5 or AKAP12 (or both) was (were) applied to the Sephacryl-400 gel filtration column (HiPrep Sephacryl S-400 High resolution 16/60, GE Healthcare) making use of a fast-performance liquid chromatography system AKTA (GE Healthcare), pre-equilibrated with PBS supplemented with 0.01% NaN_3_. Sample (1.0 ml) fractions were collected. Each fraction was analyzed by SDS-PAGE and immunoblotting. Protein was detected in the flow-cell by measuring absorbance at A280. The column was calibrated with gel filtration calibration kit HMW (GE Healthcare).

### Pull-down assay

Typically a 20 μl of AKAP12 fragment (840-1782)-Sepharose 4B or AKAP12 (1-840)-Sepharose 4B beads were incubated with purified His-tagged AKAP5 or GST-G3BP1 in phosphate buffered saline (PBS) containing 1% Triton X-100 and 1 mg/ml BSA at 4°C for 1 h. Pellets were washed with PBS containing 1% Triton X-100 three times and boiled with 30 μl Laemmli buffer at 95°C for 5 min. The supernatant was loaded onto a 7% polyacrylamide gel and subjected to sodium dodecylsulfate-polyacrylamide gel electrophoresis (SDS-PAGE). AKAP5 was detected by immunoblotting with anti-AKAP5 antibody.

### Immunoprecipitation and immunoblotting

Cells were harvested in a lysis buffer containing 20 mM Tris-HCl, pH 7.4, 1% Nonidet P-40, 2 mM sodium orthovanadate, 150 mM NaCl, 5 mM EDTA, 50 mM NaF, 40 mM sodium pyrophosphate, 50 mM KH_2_PO_4_, 10 mM sodium molybdate, and a cocktail of protease inhibitors (Complete Protease Inhibitor Cocktail tablet, Roche, Nutley, NJ). After centrifugation at 10,000 × *g *for 15 min, the protein concentration of the supernatant was determined using the Bradford reagent. One mg of protein was incubated with specific primary antibody for 4 h at 4°C, then 20 μl of protein A/G agarose was added and the mixture was incubated for 2 h on a rolling mixer. Immune complexes were collected and washed three times with PBS containing 1% Triton X-100, and thereafter boiled for 5 min at 95°C in 30 μl of Laemmli buffer. The supernatant was subjected to SDS-PAGE and the resolved proteins were transferred to a PVDF membrane. The blot was probed with specific antibodies. The immune complexes on the blots were made visible by staining with a horseradish peroxidase-conjugated secondary antibody in combination with the chemiluminescence reagent, followed by a brief autoradiography using Kodak BioMax MR film (Sigma-Aldrich, St. Louis, MO).

### Assay of β_2_-adrenergic receptor recycling

Using radioligand equilibrium binding assays with a cell-impermeant, tritiated antagonist ([^3^H]CGP-12177). HEK293 cells stably expressing β_2_-AR-GFP or A431cells were treated with isoproterenol for 30 min then washed free of agonist and allowed to recover for 120 min. The cells next were washed with ice-cold DMEM, and then changed to ice-cold Dulbecco's modified Eagle's medium containing 20 mM HEPES, pH 7.4, and the hydrophilic, membrane-impermeant β_2_-adrenergic antagonist [^3^H]CGP-12177 (10 nM). Non-specific binding was determined by adding 10 μM propranolol to the incubation solution. Binding was performed at 4°C for 4 h [24-26]. The cells were then washed with ice-cold PBS and collected with 1% SDS and 1% NP-40. The lysates were counted by scintillation spectroscopy.

## Results

### AKAP5 and AKPA12 hetero-oligomerization: analysis by affinity chromatography

**Figure 1:**
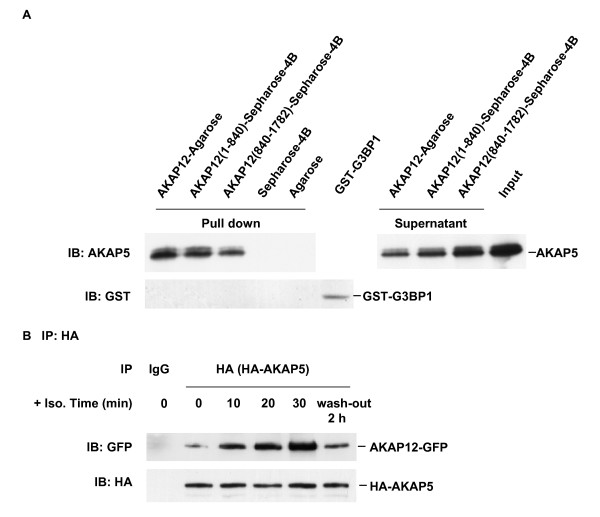
**Docking of AKAP5 to full-length AKAP12 as well as to N-(1-840) and C-(840-1782) terminal fragments**. A, Purified HA-tagged AKAP12 was immobilized to agarose beads, His-tagged AKAP12 (1-840) or His-tagged AKAP12 (840-1782) were cross-linked individually to CNBr-activated Sepharose 4B beads. Each of these bead types was incubated with either purified His-tagged AKAP5 or an equivalent amount of purified GST-tagged G3BP1 protein, as a control. At the end of the incubation the beads were collected from the mixtures and washed. The AKAP5 released from binding to sequences immobilized to the beads (Pull down), the AKAP5 input to the incubation (Input), and the AKAP5-depleted supernatants of the incubation post affinity adsorption (Supernatant) were sampled and applied to SDS-PAGE, subjected to immunoblotting and stained with anti-AKAP5 (upper panel) or anti-GST (lower panel) antibodies. B, A431 cells stably expressing GFP-tagged AKAP12 were transiently transfected with HA-tagged AKAP5. Confluent cells were treated with 10 μM isoproterenol for 10, 20, 30 min or treated for 30 min with isoproterenol and then washed-free of agonist for 2 h (wash-out 2 h). Whole-cell lysates were prepared and pull-downs conducted with anti-HA antibody (targeting HA-tagged AKAP5) or control IgG. The immune complexes were subjected to SDS-PAGE, transferred to PVDF membrane and probed with antibodies against HA tag (HA-AKAP5) or GFP (AKAP12-GFP). The results shown are taken from a single experiment, representative of at least three separate experiments performed on separate cultures of cells.

The observations that both AKAP5 and AKAP12 were capable of forming homodimers and homooligomers of higher order stimulated us to ask whether AKAPs might be able to assemble not only as homodimers/-oligomers, but also as hetero-oligomers. We probed possible interactions between these two GPCR-docking AKAPs using several different approaches. The first approach employed affinity chromatography in which AKAP12 (or a large N-terminal or C-terminal fragment) was immobilized to a solid matrix (i.e., chemically coupled to Sepharose 4B beads) and used as "bait" for AKAP5. In vitro binding assays making use of purified proteins demonstrated that the full-length AKAP12 docks AKAP5 (Figure 1A); N-terminal AKAP12 (1-840) and C-terminal AKAP12 (840-1782) also display the ability to dock AKAP5 (Figure 1A). At equivalence of derivatized matrix, the C-terminal fragment of AKAP12 bound less AKAP5 than the N-terminal AKAP12 fragment. Thus it appears that AKAP5 is capable of docking to AKAP12 either N-terminally or C-terminally to the much larger AKAP12. The matrix, absent bound either full-length or N-terminal and C-terminal fragments of AKAP12, retained no AKAP5. When an unrelated protein, GST-tagged G3BP1, was employed in place of AKAP5 in this in vitro analysis, no G3BP1 was found bound to AKAP12-derived matrices.

We next probed in vivo whether docking of AKAP5 to full length AKAP12 was influenced by activation of the A-kinase provoked via treating cells with the beta-adrenergic agonist isoproterenol. A431 cells co-expressing both HA-tagged full-length AKAP5 and GFP-tagged AKAP12 were employed. The cells expressing AKAP5 and AKAP12 were stimulated with 10 μM isoproterenol for periods of 10 to 30 min (Figure 1B). Pull-downs of HA-tagged AKAP5 were performed from whole-cell lysates and the AKAP5 analyzed for bound AKAP12. In the absence of stimulation, pull-downs of AKAP5 revealed some docking of full-length AKAP12 to AKAP5 (Figure 1B). Following stimulation with β-adrenergic agonist, the amount of AKAP12 docking to AKAP5 increased progressively over time. AKAP12 docking with AKAP5 increased up to 30 min post treatment with isoproterenol, the latest time tested. At 30 min, beta-adrenergic agonist stimulated an increase in the amount of AKAP12 docking to AKAP5 of 4-fold. We ascertained if the process was dynamic, probing reversibility of the AKAP5-AKAP12 binding once the stimulated cells were washed free of β-adrenergic agonist (Figure 1B). At 30 min of β-adrenergic agonist, the bulk of β_2_-adrenergic receptors have been fully activated, desensitized, and internalized [16,24-26]. At 2 h post wash-out, resensitization/recycling of β_2_-adrenergic receptors, a process in which AKAP12 is essential [17,24], has been completed [16,24-26]. Following the two-hour wash-out of agonist, the amount of AKAP12 found associated with AKAP5 declined sharply to the level close to the basal state. Thus AKAP12 docking to AKAP5 is enhanced by stimulation of β-adrenergic pathway and is dynamic, i.e., capable of reversal by agonist wash-out and a 2 h period of recovery in vivo (Figure 1B). Control pull-downs conducted with IgG rather than anti-HA antibody failed to reveal either bound AKAP5 or bound AKAP12.

**Figure 2:**
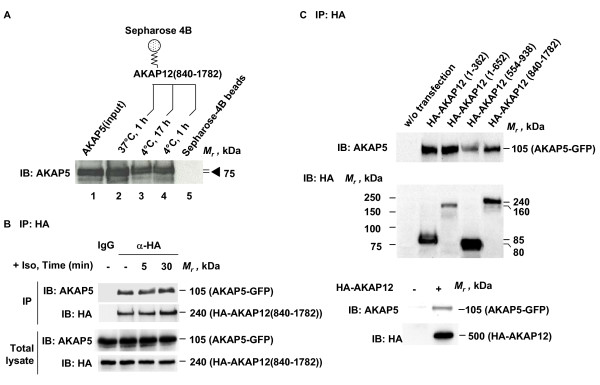
**Docking of AKAP5 to AKAP12**. A, Purified His-tagged AKAP12 (840-1782) was cross-linked to CNBr-activated Sepharose 4B, then incubated with purified His-tagged AKAP5 under the conditions labeled on each lane: lane 1, purified AKAP5, input; lane 2, pull-down at 37°C, 1 h; lane 3, pull-down at 4°C, 17 h; lane 4, pull-down at 4°C, 1 h; and lane 5, pull-down control, Sepharose 4B beads alone. B, A431 clones stably expressing GFP-tagged AKAP5 were transiently transfected to express HA-tagged AKAP12 C-(840-1782) terminal fragment. Confluent cells were treated with 10 μM isoproterenol for the indicated times. Whole-cell lysates were prepared and pull-downs performed with antibodies against the HA tag which were covalently linked to protein A/G-agarose beads or control IgG. The immune complexes were subjected to SDS-PAGE, transferred to PVDF membrane, and probed with antibodies against either AKAP5 or the HA tag. C, A431 cells stably expressing GFP-tagged AKAP5 were transiently transfected with HA-tagged AKAP12 or the AKAP12 fragments listed (i.e., 1-362; 1-652; 654-938; and 840-1782). The whole-cell lysates were prepared and pull-downs performed with antibodies against the HA tag which were covalently linked to protein A/G-agarose beads. The immune complexes were subjected to SDS-PAGE, transferred to PVDF membrane, and probed with antibodies against either AKAP5 or the HA tag. The results shown are taken from a single experiment, representative of at least three separate experiments performed on separate cultures of cells.

The ability of AKAP5 to dock to immobilized C-terminal fragment of AKAP12 (840-1782) in vitro (Figure 1A) stimulated further analysis of this docking (Figure 2A). Binding of AKAP5 to C-terminal AKAP12 was assessed by sedimentation of the Sepharose beads to which AKAP12 (840-1782) fragment was immobilized. The conditions employed for the incubation of AKAP5 with C-terminal AKAP12 beads varied from 1 h at 37°C, to overnight (17 h) and 1 h at 4°C. Purified AKAP5 displays robust docking to the C-terminal fragment of AKAP12, under each of the conditions employed. The analysis then was performed in vivo, testing AKAP5 and AKAP12 docking properties under physiological conditions. A431 cells stably expressing GFP-tagged AKAP5 were transiently transfected to express the HA-tagged AKAP12 (840-1782) fragment (Figure 2B). Pull-downs of HA-tagged AKAP12 (840-1782) performed from whole-cell lysates were quantitative. AKAP5 was shown to be capable of docking to the AKAP12 fragment in the basal, "unstimulated" state. We probed if stimulating the cells with the β-adrenergic agonist isoproterenol (10 μM) would alter the docking of AKAP5 to the AKAP12 fragment, performed under conditions in which the HA-tagged AKAP12 fragment was pulled-down and AKAP5 docking probed. β-adrenergic stimulation did not change the amount of the AKAP5 docking to the C-terminal region of AKAP12 (Figure 2B). The amounts of AKAP5 and AKAP12 fragment found in whole-cell lysates were unaffected by treating the cells with isoproterenol for up to 30 min.

To provide insight in what region AKAP5 was docking to AKAP12, we prepared various HA-tagged AKAP12 fragments (i.e., 1-362, 1-652, 554-938, and 840-1782), expressed them in A431 cells, and studied AKAP5 docking capacity of each fragment. An HA-tagged AKAP12 fragment and GFP-tagged AKAP5 were co-expressed in the cells examined. Pull-downs of HA-tagged fragments were followed by immunoblotting analysis of GFP-tagged AKAP5. Each of the AKAP12 fragments displayed some capability to bind AKAP5 (Figure 2C). The capacities of the AKAP12 fragments to dock AKAP5 varied. The fragment expressed at the highest relative abundance in cells, AKAP (554-938) displays the least docking capacity for AKAP5 of the fragments tested. AKAP5 docking to AKAP12 (1-362), the smallest fragment tested, was prominent also (Figure 2C).

### AKAP5-AKAP12 hetero-oligomerization: analysis by steric-exclusion chromatography

**Figure 3:**
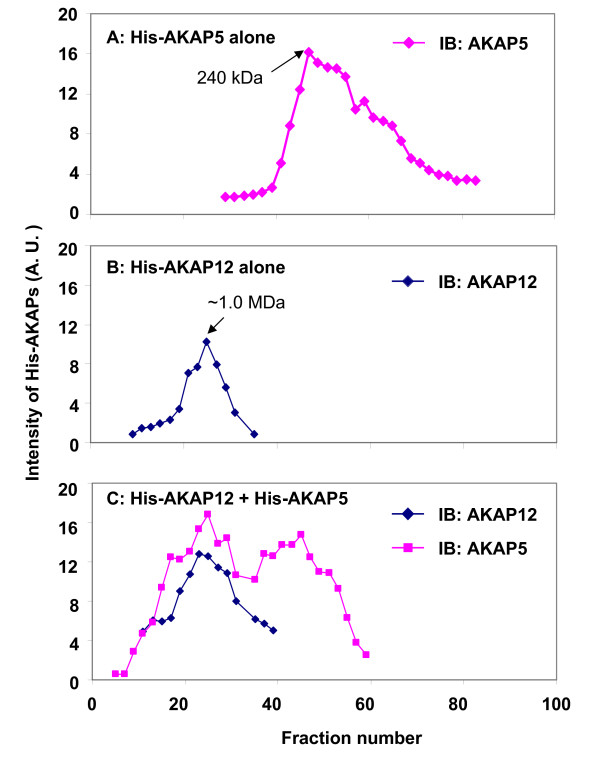
**Steric-exclusion chromatography of AKAP5 and AKAP12 alone and in combination: identification of supermolecular AKAP5/AKAP12 hetero-oligomers**. To ascertain the possible presence of higher-order oligomers of AKAP12 and AKAP5, steric-exclusion chromatography of purified AKAP5 and AKAP12 individually (A and B, respectively) or after incubation in combination (C) was performed on an AKTA FPLC (GE Healthcare) fitted with a HiPrep Sephacryl S-400 High resolution 16/60 column (GE healthcare). Marker proteins mobilities were employed to establish the MW of AKAP5-, AKAP12-, and AKAP5/AKAP12 supermolecular complexes by elution position. The A280 absorbance was monitored in real time. Samples from each fraction were subjected to SDS-PAGE and immunoblotting and staining for the AKAP12 or AKAP5. The results displayed are representative of at least three separate experiments performed on as many individual protein preparations.

We made use of wide-bore matrix steric-exclusion chromatography (SEC) to analyze the MW of oligomers of purified His-tagged versions of AKAP5 and AKAP12, both individually (Figure 3A, B; respectively), as well as following their mixing at molar equivalence (Figure 3C). A 60 cm column packed commercially with Sephacryl-400 was employed. As noted earlier, AKAP5 (MW of 427 amino acid scaffold: 47.1 kDa) forms homo-oligomers that have been reported to range from ~200 to 240 kDa-MW [28,29]. In the current experiments, AKAP5 oligomers migrated with MW of 240 kDa (Figure 3A). Under these chromatography conditions, AKAP5 oligomers do not display a sharp, symmetrical peak. Rather, the leading edge of the AKAP5 peak is sharp, but the tailing edge is broad, reflecting either dissociation of oligomers during the steric-exclusion chromatography or the presence of oligomers of non-uniform stoichiometry. A peak of monomeric AKAP5 was not apparent. The limit MW of 240 kDa suggests the presence of a tetrameric form of the purified AKAP5.

SEC analysis of purified AKAP12 (MW of 1782 amino acid scaffold: 191 kDa) displays a symmetrical peak with MW of ~1.0 MDa (MDa = Mega Dalton, Figure 3B). This higher mass MW of AKAP12 oligomer suggested the possibility of the presence of four scaffold molecules in a single supermolecular complex. The symmetry of the AKAP12 peak subjected to SEC likely reflects the marked stability of the homo-oligomeric interactions of this scaffold molecule that require 8 M urea to provoke dissociation. Although the calibration of the wide-bore Sephacryl S400 matrix is reported to provide over the range of 0.02 to 8 MDa limit size, high precision assignment of MW in this area of the chromatogram is not possible

We sought to explore if AKAP5 and AKAP12, whose sequences are in large part natively unordered [10,22], interact when incubated in combination. AKAP5 and AKAP12 were incubated together at molar equivalence (established by quantification of the His-tag) and then subjected to SEC (Figure 3C). The results of the analysis reveal a marked shift to higher mass of AKAP5. The AKAP5 profile appears to display two peaks, one with MW of ~240 kDa, as observed for the AKAP5 oligomers alone (compare Figure 3A and 3C) the other displays MW of 1-2 MDa (Figure 3C). The AKAP5 lower field ~240 kDa MW peak observed in SEC of the AKAP5/AKAP12 mixture differs from that of AKAP5 alone in a very important way. The AKAP5 lower field peak no longer displays the broad tail that extended for AKAP5 alone to the limit of the AKAP5 monomer. The SEC results suggest that in the presence of AKAP12, some AKAP5 homo-oligomeric interactions may be stabilized.

### Functional analysis of AKAP5-AKAP12 interactions

With the evidence that AKAP5 and AKAP12 forming hetero-oligomeric, supermolecular complexes, an attempt was made to probe AKAP function to see if altering the expression of one AKAP might influence the function of the other. For AKAP5 function we made use its well known role in mediating the ability of β-adrenergic receptors to activate mitogen-activated protein kinases (MAPK). The MAPKs Erk1 and Erk2 (Erk1/2) are activated in response to beta-adrenergic agonist (e.g., isoproterenol), this activation requires AKAP5, but not AKAP12 [13,23,24]. We measured the activation of ERK by immunoblotting of blots from samples of whole-cell extract subjected to SDS-polyacrylamide gel electrophoresis with phospho- and activation-specific antibody for Erk1/2.

**Figure 4:**
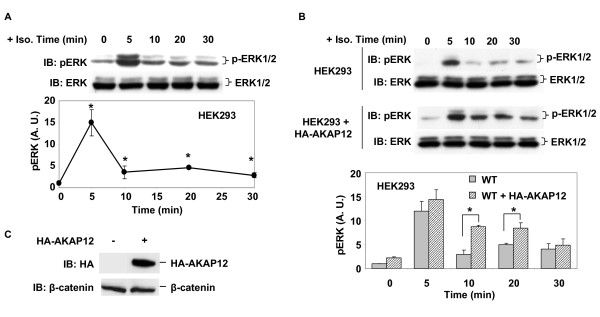
**Expression of AKAP12 potentiates Erk1/2 activation by beta-adrenergic agonist in HEK293 cells**. The cells were transiently transfected with HA-AKAP12 for 42 h and then changed to serum-free medium for 4 h. A, time course of ERK phosphorylation in HEK293 cells stimulated with 10 μM isoproterenol. B, overexpression of HA-AKAP12 potentiates ERK activation in HEK293 cells stimulated in response to 10 μM isoproterenol. C, Immunoblot analysis of expression of HA-AKAP12 using β-catenin as loading index. The blots shown are taken from a single experiment, representative of at least three separate experiments performed on separate cultures of cells. Tabular data from separate experiments was analyzed for variance using the Student's "t" test for significance. * *p *< 0.05.

HEK293 cells demonstrated a rapid, robust activation of the MAPK cascade in response to beta-adrenergic stimulation (10 μM isoproterenol), as measured by Erk1/2 activation. Within 5 min of challenge with isoproterenol, the levels of phosphorylated, activated-Erk1/2 in cells increased by more than 15-fold (Figure 4A). The 5-min sampling, the earliest time point measured, displayed the peak of the Erk1/2 activation in response to β-adrenergic stimulation. Erk1/2 activation thereafter declined to 4- to 5-fold over basal within 10 min of the stimulation. Erk1/2 activity remained slightly elevated for the remaining 20 min of the time-course following isoproterenol (10 μM) treatment. Expression of exogenous full-length, HA-tagged AKAP12 (HA-AKAP12) in the HEK293 cells potentiated the activation of Erk1/2 in response to isoproterenol (Figure 4B). At the 10 min time point following stimulation with isoproterenol, the overexpression of AKAP12 more than doubled the activation of Erk1/2 observed. The character of the time-course of Erk1/2 activation in response to β-adrenergic stimulation in HEK293 cells, potentiated by overexpression of AKAP12, was not changed (Figure 4B). Transfection with empty vector, in contrast, had no effect on the Erk1/2 activation profile in response to isoproterenol (data not shown).

**Figure 5:**
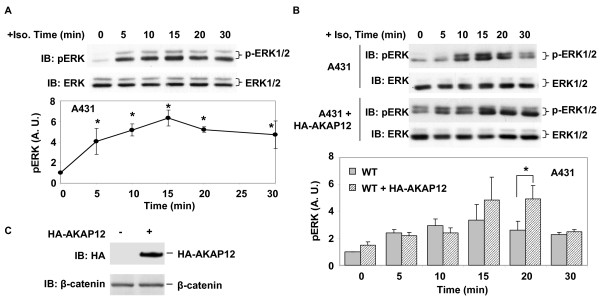
**Expression of AKAP12 potentiates activation of Erk1/2 by beta-adrenergic agonist in A431 cells**. A, time course of ERK phosphorylation in A431 cells stimulated with 10 μM isoproterenol. B, overexpression of HA-AKAP12 potentiates ERK activation in A431 cells stimulated with 10 μM isoproterenol. C, immunoblot analysis of expression of HA-AKAP12. The blots shown are taken from a single experiment, representative of at least three separate experiments performed on separate cultures of cells. Tabular data from separate experiments was analyzed for variance using the Student's "t" test for significance. * *p *< 0.05.

Erk1/2 activation by β-adrenergic agonist in other mammalian cell lines also is dependent upon on AKAP5. Specific knock-down of AKAP5 expression with siRNA reagents abolishes the ability of β-adrenergic agonists to stimulate the Erk1/2 activation response [23,24]. We extended the studies from HEK293 cells to human epidermoid carcinoma A431 cells, again probing if overexpression of AKAP12 would impact the AKAP5-mediated Erk1/2 activation in response to β-adrenergic agonist. In wild-type A431 cells, isoproterenol treatment provoked Erk1/2 activation (Figure 5A). The time-course for Erk1/2 activation by isoproterenol in A431 cells was neither as early nor as robust as that observed in HEK293 cells (compare Figures 4A, 5A). In A431 cells, peak activation of Erk1/2 occurred by 15 min post isoproterenol; whereas in HEK293 cells the peak response occurred at 5 min. In A431 cells, Erk1/2 activation in response to isoproterenol was more long-lived, remaining robust for at least 30 min (Figure 5A); whereas in HEK293 the early peak of activation declined to baseline within 10 min of the initial stimulation by isoproterenol (Figure 4A). The overexpression of HA-tagged AKAP12 potentiated the activation of Erk1/2 in response to isoproterenol in A431 cells (Figure 5B), extending similar results obtained in HEK293 cells (Figure 4B). Although the time-course of the Erk1/2 potentiation differs between the two cell lines, expression of AKAP12 clearly provoked a potentiation of the Erk1/2 response, a signaling pathway mediated via AKAP5 [23]. Transfection of empty vector, in contrast, had no effects on Erk activation (data no shown). Thus, overexpression of AKAP12, which can form hetero-oliogomeric supermolecular complexes with AKAP5, can influence the function of AKAP5.

**Figure 6:**
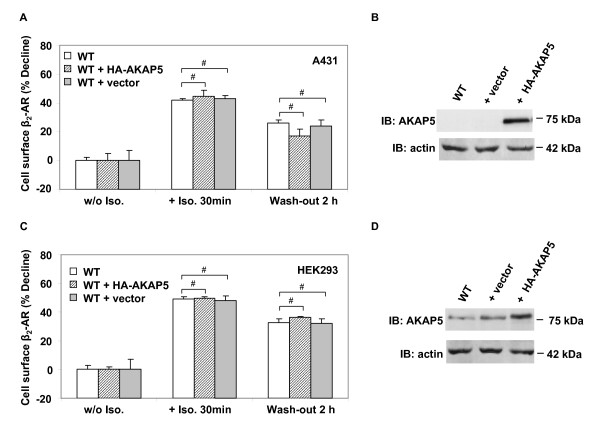
**Effects of overexpression of AKAP5 on AKAP12-mediated recycling of desensitized, internalized **β**_2_-adrenergic receptors**. HEK293 cells stably expressing β_2_-AR-GFP or wild-type A431 cells were transiently transfected with an expression vector harboring HA-AKAP5 or the empty vector for 24 h. The cells were untreated or treated with 10 μM isoproterenol for 30 min and then washed free of isoproterenol and allowed to recover for 2 h. Cell surface β_2_-AR content was detected with the cell impermeant radiolabeled beta-adrenergic antagonist [^3^H]-CGP12177. A, β_2_-AR recycling in A431 cells: effects of AKAP5 expression. B, expression of HA-AKAP5 in A431 cells analyzed by immunoblotting and staining with anti-AKAP5 antibody. C, β_2_-AR recycling in HEK293 cells: effects of AKAP5 expression. D, expression of HA-AKAP5 in HEK293 cells analyzed by immunoblotting and staining with anti-AKAP5 antibody. The results displayed are mean values ± SD derived of triplicates. For panels B and D, the results shown are taken from a single experiment, representative of at least three separate experiments performed on separate cultures of cells. Tabular data from separate experiments was analyzed for variance using the Student's "t" test for significance. # denotes *p *values > 0.05, i.e. differences that are not statistically significant.

We turned our attention to exploring if overexpression of AKAP5 might impact on a function of AKAP12. AKAP12 plays an essential role in the resensitization/recycling of desensitized/internalized GPCRs during recovery of the cell signaling pathway [3,7,9,10,27,33,34]. Knock-out of AKAP12 expression effectively abolishes the receptor resensitization and recycling. We made use of the recycling assay of desensitized/internalized β_2_-adrenergic receptors as an assay for AKAP12 function. Recycling of the receptors can be readily detected by ligand binding assays performed in whole cells using a membrane-impermeable radioligand ([^3^H]-CGP12177) that reports only the cell-surface complement of beta-adrenergic receptors [35]. Following agonist-induced receptor internalization, AKAP12 catatlyzes the recycling of the receptor back to the cell surface where they can bind CGP12177 ligand. Exogenous, HA-tagged AKAP5 was expressed in A431 as well as in HEK293 cells (Figure 6). The low levels of endogenous β_2_-adrenergic receptors in HEK293 cells required exogenous expression to accommodate the recycling assay; HEK293 clones stably expressing GFP-tagged β_2_-adrenergic receptors were employed for these studies. The AKAP12-mediated recycling of internalized β_2_-adrenergic receptors was assessed in untreated cells (w/o Iso), isoproterenol-treated cells (+Iso, 30 min), and in cells treated with isoproterenol and then washed free of agonist for 2 h (Wash-out 2 h, see Figure 6A, C). The assay results are reported in units of "%-decline" of cell surface β_2_AR. AKAP5 was readily expressed in the A431 and HEK293 cells, but in neither cell line was the AKAP12-catalyzed recycling of β_2_-adrenergic receptors altered by overexpression of AKAP5. The amount of recycling of the desensitized, internalized beta2-adrenergic receptor at 2 h post agonist wash-out (i.e., at peak recovery and recycling) was the same for the wild-type cells/clones as for those overexpression AKAP5.

## Discussion

This work establishes a new and important observation in cell signaling, i.e., hetero-oligomerization of AKAPs. Earlier AKAP5 and AKAP12 were shown to form homo-dimers and homo-oligomers [28,29]. AKAP12 molecules were found to be so avidly bound to each other that only the presence of the strong chaotropic agent (e.g., 8 M urea) disrupted the oligomers to monomers. The existence of the more weakly associating homo-oligomers of AKAP5, in contrast, could be revealed only through affinity pull-downs (e.g., co-immunoprecipitation) and by steric-exclusion chromatography [28]. We show herein that AKAP5 and AKAP12 display the ability to hetero-oligomerize, forming very large (1-2 MDa MW by SEC sieving) supermolecular complexes. The presence of hetero- as well as homo-oligomers suggests the possibility of the existence of higher-order complexes among members of the wider AKAP family. Such higher order of protein-protein interactions may offer additional dynamic character to signaling molecules that dock to each of these AKAP scaffold complexes, *e.g*., including GPCRs [10,21,27], serine/threonine protein kinases [3,4,15,16], tyrosine kinases [9], phosphoprotein phosphatases [7], cyclic AMP phosphodiesterases (e.g., PDE4) [11-14], as well as other docking proteins [3,4,36].

Another new dimension of AKAP biology is that the docking of the mobile AKAP12 to the AKAP5 bound to the cell membrane is shown to be sensitive to activation of the cyclic AMP pathway, provoking an intimately orchestrated spatio-temporal association of these two scaffolds. Uncovering the functional implications of AKAP-AKAP hetero-oligomerization, if any, was an important task. Overexpression of AKAP12 is shown to markedly potentiate the ability of AKAP5 to mediate the β-adrenergic agonist regulation of Erk1/2 activation. In both HEK293 and A431 cells, overexpression of AKAP12 potentiated AKAP5-mediated signaling. The increased AKAP12, if docking to AKAP5, might recruit additional signaling molecules to the locale membrane microenvironment. These molecules could stabilize the beta-adrenergic stimulus and thereby potentiate the activation of Erk1/2. On the other hand, we did not see any detectable effect of AKAP5 overexpression on AKAP12-mediated signaling read-outs, e.g., beta-adrenergic receptor resensitization and recycling. This observation confirms our earlier findings [23], that AKAP5 was not necessary for beta-adrenergic receptor resensitization/recycling. Knock-down of AKAP5 did not significantly affect AKAP12-mediated receptor recycling. AKAP5 localizes strictly to the cell membrane, which would have a nearly infinite capacity to bind this scaffold, even when overexpressed. In this feature, AKAP5 is fundamentally unlike AKAP12 which can be found throughout the cytoplasm and dynamically docks to the cell membrane.

Full-length AKAP12 docks to the membrane-bound AKAP5. Full-length AKAP12 as well as the large N- and C- terminal fragments of AKAP12 displayed the capacity to dock to AKAP5. A large central region (556-938) of AKAP12, in contrast, displayed the least capacity to dock AKAP5. This same region of AKAP12 was shown earlier to be essential for AKAP12 function, itself phosphorylated by protein kinase A in response to beta-adrenergic agonist treatment [27]. Further, this region harbors the Receptor-Binding Domain (RBD), which interacts reversibly with β-adrenergic receptors (and perhaps other GPCRs) during the desensitization/internalization and resensitization/recycling of the GPCR to the cell membrane. A domain with analogy to the RBD, which also is a substrate for protein kinase A-catalyzed phosphorylation, can be discerned in AKAP5; both AKAP5 and AKAP12 dock β-adrenergic receptors [10,22].

**Figure 7:**
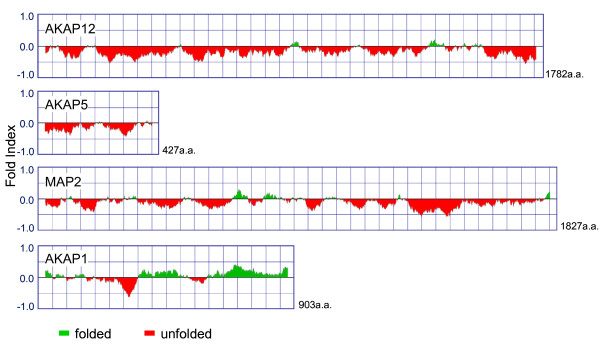
**FOLDIndex^© ^analysis of AKAP family members AKAP12, AKAP5, MAP2, and AKAP1: degree of natively "unordered" structure**. The primary sequences of human AKAP5, AKAP12, and several other members of the AKAP family were analyzed for natively ordered structure by application of the FOLDindex^©^. The sequences are displayed from N-terminus to C-terminus (left to right) for four of the AKAP sequences selected. The regions below the horizontal midline, filled in with red, are predicted to be natively unfolded based upon primary sequence information alone. The regions above the horizontal midline, filled in with green, are predicted to be natively folded, based upon primary sequence.

Uncovering the molecular basis for AKAP oligomerization remains an important target. The primary sequences of both AKAP5 and AKAP12, although clearly replete in docking sites for numerous proteins, are predicted to be largely "natively-unordered" (Figure 7). FOLDindex^© ^predicts intrinsic unfolding based upon the average residue hydrophobicity and the net charge in the primary sequence of a protein [37,38]. By FOLDindex^©^, human AKAP12 is predicted to be >90% natively unordered, whereas AKAP5 is predicted to be >80% natively unordered. Intuitively, we might suspect that a high content of unordered structure would preclude or at least limit the function of a scaffold (i.e., unordered sequences would seem to afford fewer docking sites for the large number of protein ligands interacting with AKAP12) [10,22,26]. Not only AKAP5 and AKAP12, but also MAP2 and other AKAP family members, by this same analysis, appear to be largely natively unordered. AKAP1, in contrast to AKAP12, AKAP5, or MAP2, does display significant regions of ordered structure (Figure 7). By analogy, Dishevelled-1, -2, and -3, are scaffold proteins that function in Wnt signaling. Each of these Dishevelleds is predicted by FOLDindex to possess >85% ordered structure [39,40], including the presence of DIX, PDZ, DEP domains and sites for a multitude of docking partners [39].

We speculate that the "natively unordered" primary sequence of AKAPs, like AKAP5 and AKAP12, constitute docking sites quite different from highly-structured domains such as DIX, PDZ, and DEP. The high content of natively unordered sequence (and prolyl residues) must provide some additional "flexibility" or benefit that cannot be deduced solely by analysis of the primary sequence by the FOLDindex^©^. AKAP12, as noted above, displays a Receptor-Binding Domain for GPCRs [27], several docking sites for protein kinase C [33,34], and for PDE4 [14] in just the very regions that are reported to be largely "natively unordered". More detailed analysis of sites of homo-oligomerization as well as of docking sites involved in AKAP hetero-oligomerization is essential. Establishing the basis for the oligomerization of AKAP monomers to higher order, supermolecular hetero-oligomers in response to activation of beta-adrenergic pathway will be required to more fully understand the biology of AKAP scaffolds in cell signaling.

## Conclusion

The current investigation demonstrates for the first time that members of the AKAP family, AKAP5 and AKAP12, can form not only homo-oligomers, but also hetero-oligomers. The hetero-oligomeric AKAP are higher-order supermolecular complexes. The intracellular ratio between AKAP5/AKAP12 can influence functions of AKAPs. Thus, the existence of large, supermolecular hetero-oligomers of AKAPs adds a new dimension to the possibilities for docking of one AKAP with another member of the AKAP family in novel and functionally important ways.

## Abbreviations

AKAP: A-kinase Anchoring Protein; DMEM: Dulbecco's modified Eagle's medium; A431: human epidermoid carcinoma cells; HEK 293: human embryonic kidney 293 cells; SDS-PAGE: sodium dodecyl sulfate-polyacrylamide gel electrophoresis; SEC: steric-exclusion chromatography.

## Competing interests

The authors declare that they have no competing interests.

## Authors' contributions

The authors contributed equally to the evolution of the study design; the authors contributed equally, designed/performed the studies, gathered the data, and outlined a draft of the manuscript. SG collected the data and wrote the draft manuscript; HYW and CCM edited the draft manuscript and figures of the final version of this unpublished work. Each author read and approved the final manuscript.
